# Controlling a complex system near its critical point via temporal correlations

**DOI:** 10.1038/s41598-020-69154-0

**Published:** 2020-07-22

**Authors:** Dante R. Chialvo, Sergio A. Cannas, Tomás S. Grigera, Daniel A. Martin, Dietmar Plenz

**Affiliations:** 1 Center for Complex Systems and Brain Sciences (CEMSC3), Escuela de Ciencia y Tecnología, Universidad Nacional de Gral. San Martín, Campus Miguelete, 25 de Mayo y Francia, 1650 San Martín, Buenos Aires Argentina; 2 Consejo Nacional de Investigaciones Científicas y Técnicas (CONICET), Godoy Cruz 2290, 1425 Buenos Aires, Argentina; 3 Instituto de Física Enrique Gaviola (IFEG-CONICET), Facultad de Matemática, Astronomía, Física y Computación, Universidad Nacional de Córdoba, 5000 Córdoba, Argentina; 4 Instituto de Física de Líquidos y Sistemas Biológicos (IFLySiB-CONICET), Universidad Nacional de La Plata, 1900 La Plata, Buenos Aires Argentina; 5 Departamento de Física, Facultad de Ciencias Exactas, Universidad Nacional de La Plata, La Plata, Argentina; 6 Section on Critical Brain Dynamics, National Institute of Mental Health, Bethesda, MD 20892 USA

**Keywords:** Computational biology and bioinformatics, Neuroscience, Systems biology, Mathematics and computing, Physics

## Abstract

Many complex systems exhibit large fluctuations both across space and over time. These fluctuations have often been linked to the presence of some kind of critical phenomena, where it is well known that the emerging correlation functions in space and time are closely related to each other. Here we test whether the time correlation properties allow systems exhibiting a phase transition to self-tune to their critical point. We describe results in three models: the 2D Ising ferromagnetic model, the 3D Vicsek flocking model and a small-world neuronal network model. We demonstrate that feedback from the autocorrelation function of the order parameter fluctuations shifts the system towards its critical point. Our results rely on universal properties of critical systems and are expected to be relevant to a variety of other settings.

The last decade has witnessed an escalating interest in complex biological phenomena at all levels including macroevoluction, neuroscience at different scales, and molecular biology. The observed complexity in nature is often traced to critical phenomena because it resembles the complexity found for critical dynamics in models and theory^1–8^. However, as discussed elsewhere, such resemblances are far from enough to attribute to criticality the mechanism behind all forms of natural complexity. Even though out of equilibrium generic scale invariance can arise without fine-tuning of control parameters^9–12^, it is often found that biological systems operate in special regions of control parameter space which are critical in the sense that they separate phases of different dynamical behavior^3^. More specifically, it seems that many biological systems reach a “sweet spot” where they attain maximal susceptibility, i.e., sensitivity to changes in the environment, while maintaining internal order. It is important to emphasize that at present it is not clear how such a critical state can be reached or even maintained. For a complex system like the brain, one might imagine that its control parameters be hard-wired genetically, selected by a long evolutionary process to a critical point that is biologically most advantageous for survival. However, the values the control parameters need to attain for the system to maximize its susceptibility *depend on system size*^13^, and thus for biological systems to take advantage of critical dynamics they would need to adjust the control parameter as systems contract or expand^14^. We exemplify this problem in Fig. 1 (inset) which sketches how the peak of the susceptibility, the property to be maximized, shifts as the system gets larger.

While some physical systems might be large enough that one can assume they are asymptotically near the thermodynamic limit, we note that most biological systems are of moderate size, and finite-size effects are in principle to be expected^14^. It has been argued that the critical point is the best (or only) functioning state for a given biological system. In order to attain it, Darwinian evolution instead of furnishing a set of specific values for the control parameter must allow for a *control mechanism* such that systems can reach and stay close to a critical point.

We show that a system can be tuned to the vicinity of its finite-size “critical” (i.e. maximum susceptibility) point using the first autocorrelation coefficient *AC*(1) of the order parameter fluctuations (we define *AC*(1) as in time-series analysis as the time correlation of the order parameter at $t=1$). Because *AC*(1) peaks at the same point as the susceptibility, yet does so more smoothly than the susceptibility, a control feedback seems straightforward. This behavior of *AC*(1) near criticality responds to the notion of critical slowing down in both equilibrium and non-equilibrium critical dynamics by which perturbations take longer to dissipate near criticality.

The existence of the maximum of *AC*(1) can be understood from dynamic scaling. The dynamic scaling form of the time correlation is^15^

1 $$\begin{aligned} C(k,t) = C_0(k) g\left( \frac{t}{\tau _0(k,\xi )}; k\xi \right) , \end{aligned}$$

where *k* is the observation wavevector, $\xi$ is the correlation length, the function *g* is such that $g(t=0)=1$, i.e. $C_0(k)$ is the static correlation function, and the characteristic time obeys

2 $$\begin{aligned} \tau _0(k,\xi ) = k^{-z} \Gamma (k\xi ;\xi ) = \xi ^z \Omega (k\xi ), \end{aligned}$$

where *g*, $\Gamma$ and $\Omega$ are unspecified scaling functions and *z* is the dynamic scaling exponent. Now

3 $$\begin{aligned} \frac{C(k,t=\delta t)}{C(k,0)}\approx & {} 1 + \delta t \frac{1}{C_0(k)} \frac{dC(k,t=0)}{dt} \nonumber \\&= 1 + \delta t\xi ^{-z} \Omega ^{-1}(k\xi ) g'(0;k\xi ). \end{aligned}$$

For a global quantity, $k=0$ (see Suppl. Material) so that

4 $$\begin{aligned} \frac{C(k=0,\delta t)}{C(k=0,t=0)} \sim 1- A\, \xi ^{-z} \sim 1- A\, (T-T_c)^{z \nu }, \end{aligned}$$

where $A>0$ is a time dependent constant. Hence, the normalized time correlation has a maximum at $T=T_c$, for fixed $\delta t$.

**Figure 1:**
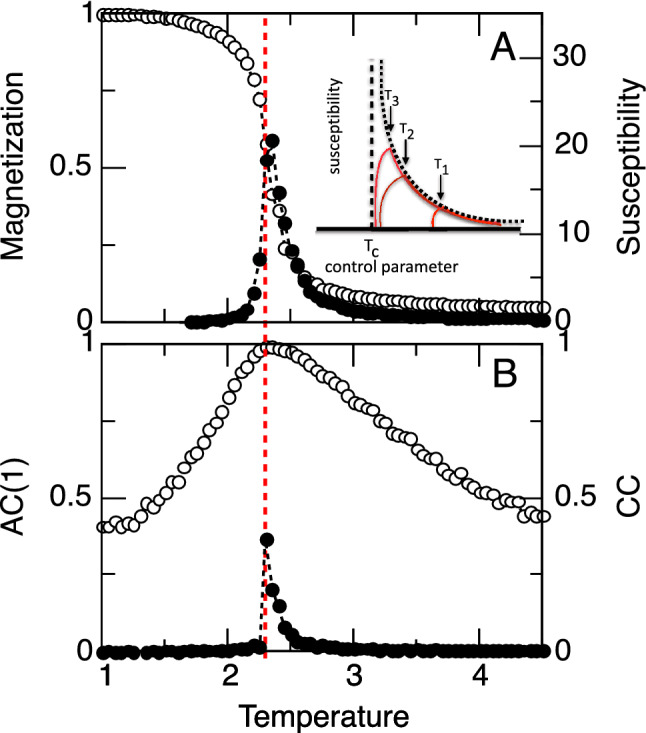
Autocorrelation peaks with susceptibility at $T_c$ in the equilibrium Ising model. Panel **A**: The order parameter (magnetization; open circles) and susceptibility (filled circles) as function of temperature *T*. Panel **B**: Corresponding average pairwise correlation (*CC*; filled circles) and first autocorrelation coefficient (*AC*(1); open circles) of the magnetization fluctuations around the instantaneous mean. Dashed vertical line denotes $T_c$. (System size $N=32^2$, $10^4$ MC steps). The inset shows a cartoon of the expected susceptibility as a function of the control parameter for three systems of increasing sizes, where arrows indicate the corresponding optimal points $T_1, T_2,T_3$.

The main idea is demonstrated here by applying it to three well understood systems, namely the ferromagnetic Ising model, the Vicsek model of flocking and a typical neuronal small-world network. We remark that the results are general enough to be also expected in many other systems.

**Ising model** Figure 1 illustrates the typical behaviour of the 2D ferromagnetic Ising model at increasing temperatures. The system undergoes a second order phase transition at a critical temperature $T_c$, reflected in a steep change in magnetization as well as a sharp peak in susceptibility (Fig. 1A). Equally distinct changes are also demonstrated for the correlation properties of the model computed from appropriate system variables (Fig. 1B). A sharp increase in the average pairwise correlations is observed as the system approaches $T_c$, where the correlation length matches the size of the system. The relatively sharp changes in the spatial correlations contrast with the relatively smoother changes in the temporal correlations, as reflected by the first auto-correlation coefficient *AC*(1) of the magnetization fluctuations around the mean, which at $T_c$ approaches unity.

Now we asses how to control the Ising model to stay at the vicinity of the susceptibility peak. According with the discussion in the introduction, we must restrict ourselves to do it using only either local or global information. In that sense, the time correlation evaluated by *AC*(1) meets such conditions, because it can be computed from a temporally delayed version of a global average of magnetization. In turn, magnetization can be assessed simply by averaging samples of a relatively large number of sites.

**Figure 2:**
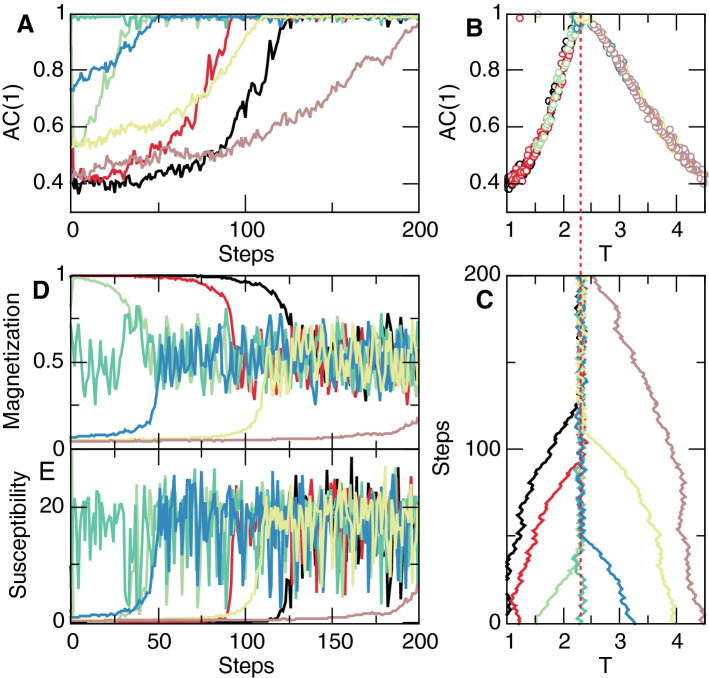
Adaptive control of the Ising model with temperature adjusted iteratively by the autocorrelation of the order parameter. The data illustrate, for a variety of initial temperatures, the convergence of the system to the vicinity of the expected critical temperature $T_c=2.3$. Panels **A**, **C**, **D**, **E** show, as a function of iteration steps, the first autocorrelation coefficient of the magnetization’ fluctuations, the magnetization, the susceptibility, and the temperature. As seen in Panel **C**, for any initial temperature the system converges to values near the equilibrium $T_c$ and at the maximum of *AC*(1) (see panel **A**). Adaptation parameter $\kappa =0.04$, other parameters as in Fig. 1. Colors denote the evolution of variables toward the critical point starting from five different initial conditions of temperature *T*: 0.5 (black), 0.1 (red), 0.16 (light green), 0.2 (dark green) and 0.35 (blue).

To demonstrate control we proceed by choosing an initial random temperature and simulate the dynamics for some large number of Montecarlo (MC) steps, which we denote as an “adaptation iteration step” indexed by *i*. We proceed by estimating the *AC*(1) of the fluctuations around the mean magnetization during the lapse of time corresponding to the adaptation iteration step *i* and monitor the change of *AC*(1) between two consecutive steps *i*, defining

5 $$\begin{aligned} d_i= d_{(i-1)}\, \text {sign}[AC(1)_{(i)} - AC(1)_{(i-1)}], \end{aligned}$$

so that *d* changes sign when a decrease in *AC*(1) is detected. We then use the gradient to its maximum value

6 $$\begin{aligned} \delta _i=(1- AC(1)_{(i)})^2, \end{aligned}$$

to change the future temperature $T(i+1)$ (i.e., the control parameter) according to

7 $$\begin{aligned} T_{(i+1)}=T_{(i)} + \delta * d*\kappa , \end{aligned}$$

where $\kappa$ is a constant that determines how slowly the temperature is adjusted. Its exact value is not crucial for the present results. Successive iterations of Eqs. 5–7 demonstrate convergence of the temperature to the expected value at equilibrium $T_c \sim 2.3$. Fig. 2 illustrates typical results for various initial temperatures, which in all cases converge to the vicinity of $T_c$. We note that the successive values of the parameters (order, control and *AC*(1)) obtained during the adaptive simulations over-imposes well (i.e., matches) those obtained from equilibrium simulations.

**Figure 3:**
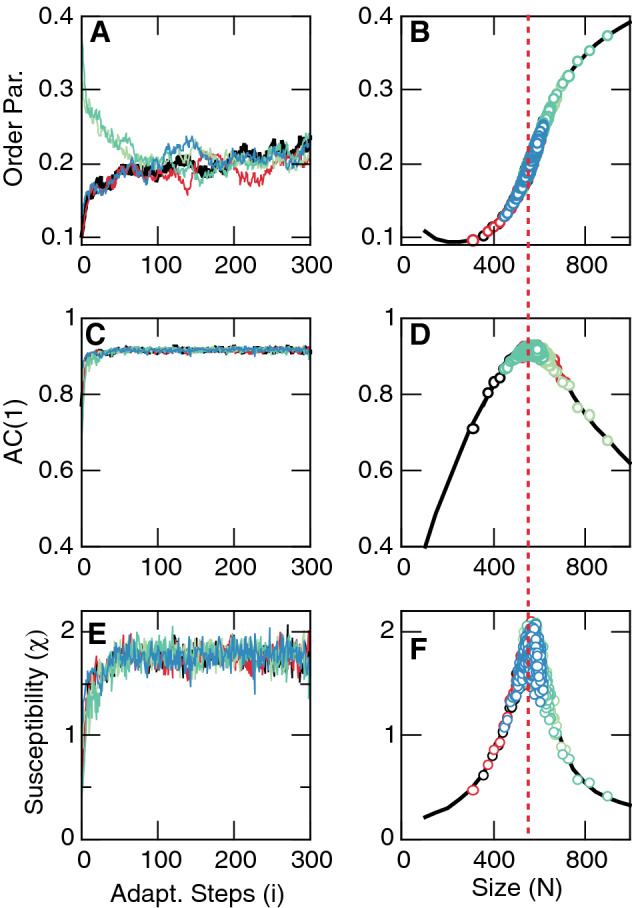
3D Vicsek model at equilibrium and under adaptive control. Order parameter $\varphi$ (Panel **A**, **B**), the first autocorrelation coefficient *AC*(1) of the polarization fluctuations around the mean (Panel **C**, **D**) and the susceptibility $\chi$ (Panel **E**, **F**) (computed as $\text {var}(\varphi )*N$) and as a function both of adaptation steps (left columns) and of the system size *N* (right columns). Notice the overlap between the equilibrium results (solid lines) and the values reached during the adaptive control (open circles) for different initial conditions which converge to the critical size $N_c\sim 560$ denoted by the dashed line. $\eta = 0.5$, $v_0=1$, $L=7.5$. $\kappa =800$ and $10^4$ MC steps per adaptation iteration step. Colors are used to identify the evolution of the variables toward the critical point, starting from five different initial conditions of size *N*: 200 (red), 300 (black), 400 (blue), 750 (light green) and 1000 (dark green).

**Figure 4:**
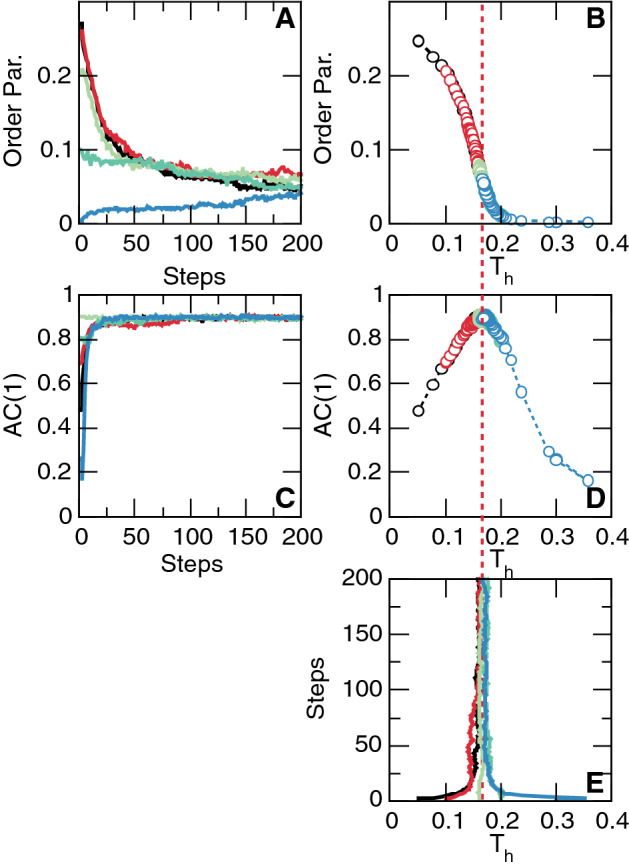
Adaptive control of the neuronal network model. Data corresponds to numerical solutions of the model starting from five different initial conditions of the control parameter $T_h$. Different colors denote the evolution of the variables towards the critical point for each initial condition $T_h$: 0.05 (black), 0.1 (red), 0.16 (light green), 0.2 (dark green) and 0.35 (blue). Nodes threshold $T_h$ are adjusted iteratively according to Eqs. 5–7. Panels **A**, **C** and **E** show the evolution of the order parameter, *AC*(1) and control parameter $T_h$ respectively. These quantities are plotted against each other in Panels **B** and **D** to demonstrate convergence to the critical value of $T_h$ (dashed red line) and to the maximum of *AC*(1). $\kappa =0.1$ and $5*10^3$ time steps per adaptation iteration step. Network parameters are: mean degree ${<}k{>} = 10$, rewiring parameter $\beta =0.3$, system size $N=2^9$. Non null weights chosen from a distribution $p(w)=\lambda \, e^{-\lambda \, w}$ with $\lambda =12.5$.

**Vicsek model** We were also able to use the *AC*(1) function to control the Vicsek model^16^, the archetypal model for flocking behavior, towards its critical point. In this model, *N* self-propelled particles endowed with a fixed speed $v_0$ move in *d*-dimensional space. At each time step, positions ${\mathbf{r}}_i(t)$ and velocities ${\mathbf{v}}_i(t)$ are updated according to

8 $$\begin{aligned} \mathbf{v_i}(t+\Delta t)&= v_0 {\mathscr{R}}_\eta \left[ \sum _{j\in S_i} {\mathbf{v}}_j(t) \right] , \end{aligned}$$

9 $$\begin{aligned} \mathbf{r_i}(t+\Delta t)&= {\mathbf{r}}_i(t) + \Delta t {\mathbf{v}}_i(t+\Delta t), \end{aligned}$$

where $S_i$ is a sphere of radius $r_c$ centered at ${\mathbf{r}}_i(t)$. The operator $R_\eta$ normalizes its argument and rotates it randomly within a spherical cone centered at it and spanning a solid angle $\eta \Omega _d$, where $\Omega _d$ is the area of the unit sphere in *d* dimensions ($\Omega _2=2\pi$, $\Omega _3=4\pi$).

The order parameter, which measures the degree of flocking, is the normalized modulus of the average velocity^16,17^,

10 $$\begin{aligned} \varphi \equiv \frac{1}{N v_0} \left| \sum _{i=1}^{N} {\mathbf{v}}_i \right| . \end{aligned}$$

$\varphi \in [0,1]$, with $\varphi = O(1/\sqrt{N})\sim 0$ in the disordered phase and $\varphi = O(1)$ in the the ordered phase. We choose $\Delta t= r_c = 1$, noise amplitude $\eta =0.5$, the speed $v_0$ and the number density $\rho = N/V$, where $V=L^d$ is the volume of the (periodic) box. Here, we choose *N* as the control parameter noting that similar results are obtained using noise amplitude $\eta$ as control parameter for fixed *N* (see Suppl. Material). We apply Eqs. 5–7 to this model, using $\eta$ as control parameter and keeping the density fixed. For comparison we over-plotted results from equilibrium runs with values taken during adaptive control of the simulations (Fig. 3). The close match demonstrates that the technique is able to control the flock model near its critical size $N \sim 560$.

**Neuronal network model** Successful control was further demonstrated for a previously described neural network model^18^ consisting of a network of interconnected nodes together with a dynamical rule. The model exhibits a second order phase transition^20^ on a region of parameters. The model matrix of interactions follows a small-world topology and each node exhibits discrete state excitable dynamics, following the Greenberg–Hastings model^19^. Briefly, each node is assigned one of three states: quiescent *Q*, excited *E*, or refractory *R*, and the transition rules are: (1) $Q \rightarrow E$ with a small probability $r_1$ ($\sim 10 ^{-3}$), or if the sum of the connection weights $w_{ij}$ with the active neighbors (*j*) is higher than a threshold $T_h$, i.e., $\sum w_{ij} > T_h$ and $Q \rightarrow Q$ otherwise; (2) $E \rightarrow R$ always; (3) $R \rightarrow Q$ with a small probability $r_2$ ($\sim 10 ^{-1}$) delaying the transition from the *R* to the *Q* state for some time steps. Parameters $r_1$ and $r_2$, which determine the time scales of self-excitation and of recovery from the excited state, respectively, were kept fixed and $T_h$ was updated according to control Eqs. (5–7). The density of excited nodes, (i.e., in state E) in each time step was taken as the order parameter. As shown for the previous models, the feedback of the *AC*(1) of that order parameter was able to move and maintain the system near its critical point (here $T_h\sim 0.16$) (Fig. 4). It is important to remark, that the neurons excitability is adjusted not by the network rate of activity, but by the temporal correlations (i.e., *AC*(1)) of such activity fluctuations.

We have verified that the present results apply, with some small differences, to systems undergoing either 1st or 2nd order phase transitions. We note also that low dimensional dynamical systems exhibiting continuous or discontinuous bifurcations from fixed points to limit cycles which can be controlled, using the same idea, near the bifurcation point.

In conclusion we have demonstrated, in three paradigmatic cases, how to shift the system towards its critical point using a feedback loop between the control parameter and the first autocorrelation coefficient of the order parameter fluctuations. Our results build at least on two previous lines of work which come close to describe this control strategy. One is the view of self-organized criticality^1^ as a feedback between order and control parameters^21^. The other line relates to forecasting of an upcoming tipping point via the generic slowing down present at criticality^22,23^. The current results go beyond these previous approaches by demonstrating an alternative mechanism for the presence of criticality in some systems. Furthermore it provides a strategy of control amenable of practical implementations in different areas. For instance, in neuroscience, this approach could be implemented in conjunction with optogenetical targeting^24^ in behaving rodents to clamp cortical networks to any desired dynamical state, helping to predict its influence on perceptual performance during a given task.

## Supplementary material

Supplementary information
