# Supplementary material for: Controlling a complex system near its critical point via temporal correlations
Source: Sci Rep. 2020 Jul 22;10:12145. doi: 10.1038/s41598-020-69154-0 (PMC7376152; doi:10.1038/s41598-020-69154-0)
Supplement: Supplementary file 1 — Supplementary information [file 41598_2020_69154_MOESM1_ESM.pdf]

Supplemental Material of the manuscript: Controlling a complex system near its critical point via temporal correlations

Dante R. Chialvo, Sergio A. Cannas, Tomás S. Grigera, Daniel A. Martin and Dietmar Plenz

FIRST AUTOCORRELATION COEFFICIENT: FINITE SIZE ANALYSIS

The first autocorrelation coefficient can be defined as $AC(1) = C_m(\delta t)$, where

$$C_m(t) = \frac{\langle m(0)\, m(t)\rangle - \langle m(0)\rangle\langle m(t)\rangle}{\langle m^2\rangle - \langle m\rangle^2} \tag{1}$$

is the autocorrelation function of the order parameter $m(t)$ and δt is a short, fixed time. Notice that $AC(1)$ is a global, not local, quantity. The term and the acronym $AC(1)$ is used here following the common practice in time-series analysis considering $m(t)$ in Eq.1 above as a time series. The autocorrelation coefficient is then reflecting here a quantity "local" in time, not space. In turn, $C_m(t)$ is related to the particle-particle correlation function $C(r,t)$ through

$$C_m(t) = \frac{\int C(r,t) d\vec{r}}{\int C(r,0) d\vec{r}}. \tag{2}$$

$C(r,t)$ is the average correlation between the state variables of two particles located at a distance r, one taken at time zero and the other at time $t > 0$. In terms of the Fourier transform $C(k,t) = \int e^{i\vec{k}.\vec{r}}\, C(r,t) d\vec{r}$, Eq.(2) reads

$$C_m(t) = \frac{C(k=0,t)}{C_0(k=0)}. \tag{3}$$

where $C_0(k) \equiv C(k, t=0)$. Then, assuming the dynamic scaling hypothesis

$$C(k,t) = C_0(k) g\left(\frac{t}{\tau_0(k,\xi)}, k\xi\right) \tag{4}$$

from equations (3) and (4) of the main manuscript we obtain (close to the critical point)

$$AC(1) \sim 1 - A\ (T - T_c)^{z\nu} \tag{5}$$

where $A > 0$ is a time dependent constant.

To take finite-size effects into account, let us go back to (4) and rewrite it as

$$C(k;t;L) = C_0(k;L/\xi) g\left(\frac{t}{\tau_0(k,\xi;L)}; k\xi; L/\xi\right). \tag{6}$$

Expanding near $t = 0$ as before,

$$1 - \frac{C(k;\delta t;L)}{C(k;t=0;L)} \approx \delta t \frac{g'(t=0;k\xi;L/\xi)}{\tau_0(k;\xi;L)}. \tag{7}$$

Setting $k = 0$ we use the finite-size scaling relation for τ [1],

$$\tau_0 = L^z \hat{\tau}(L/\xi) = L^z \hat{\tau}(L\,|T - T_c|^\nu), \qquad \hat{\tau}(x) \sim \begin{cases} \text{const} & x \to 0 \\ x^{-1} & x \to \infty \end{cases} \tag{8}$$

and then

$$1 - \frac{C(k=0;\delta t;L)}{C(k=0,t=0;L)} \approx \delta t L^{-z} g_2(L\,|T - T_c|^\nu), \tag{9}$$

where $g_2(x) = g'(t=0; k\xi=0; x)\hat{\tau}^{-1}(x)$.

Thus, from this analysis we expect i) that at $T = T_c$, $AC(1) \sim 1 - L^{-z}$, and ii) that AC(1) is closest to 1 at a temperature given by the minimum of $g_2(x)$. This gives a shift in T_c with the usual $1/\nu$ exponent: if the minimum of $g_2(x)$ is at x^*, then $T_c(L)$ is such that $L\,|T_c(L) - T_c|^\nu = x^*$, so that

$$T_c(L) = T_c + (x^*)^{1/\nu} L^{-1/\nu}. \tag{10}$$

ADAPTIVE TUNING OF THE VICSEK MODEL USING NOISE AMPLITUDE η

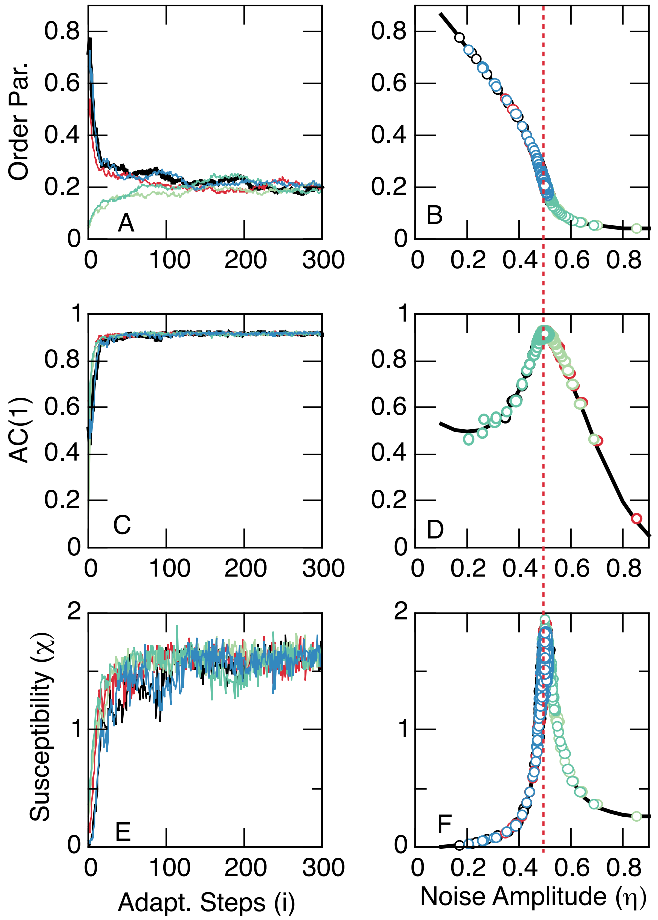

FIG. 1: Example of the 3D Vicsek model at equilibrium and under adaptive control using the noise amplitude η as the control parameter. Order parameter φ (Panel A,B), the first autocorrelation coefficient AC(1) of the polarization fluctuations around the mean (Panel C,D) and the susceptibility χ (Panel E,F) (computed as $\text{var}(\varphi) * N$) as a function both of adaptation steps (left columns) and of the noise amplitude η (right columns). Notice the overlap between the equilibrium results (solid lines) and the values reached during the adaptive control (open circles) for different initial conditions which converge to the critical noise amplitude $\eta_c \sim 0.5$ (vertical dashed line). $N = 560$, $v_0 = 1$, L=7.5. $\kappa = 0.2$ and 10^4 MC steps per adaptive iteration step. Colors are used to identify the individual evolution of the control, starting from five different initial conditions of noise amplitude η: 0.15 (black), 0.2 (blue), 0.35 (red), 0.7 (dark green) and 0.9 (light green).

[1] H. Takano, Finite Size Scaling Approach to the kinetic Ising Model, *Prog. Theor. Phys.* **68**, 493 (1982).
